# Medical errors, affected sites, and adverse consequences among patients in the orthopaedic department: Does age matter?

**DOI:** 10.3389/fpubh.2024.1306215

**Published:** 2024-02-21

**Authors:** Paicheng Liu, Jianxin Cheng, Yuxuan Yang, Haipeng Zhu

**Affiliations:** ^1^Department of Orthopaedics, Guangdong Women and Children Hospital, Guangzhou, China; ^2^School of Public Administration, Southwestern University of Finance and Economics, Chengdu, China; ^3^School of Public Administration and Emergency Management, Jinan University, Guangzhou, China; ^4^School of Government, Sun Yat-sen University, Guangzhou, China

**Keywords:** adverse consequences, affected sites, aging and public health, medical errors, orthopaedic, patient safety

## Abstract

**Background:**

Orthopaedics have become the focus of research on patient safety due to the high incidence of medical errors. Previous studies were based on all orthopaedic patients and rarely conducted empirical analyses from the perspective of age. This study aimed to fill the academic gap in the age variable by comparing medical errors, affected sites, and adverse consequences in orthopaedic patients.

**Methods:**

This retrospective study included 329 litigation claims against orthopaedists using data from China Judgments Online. First, we performed computer crawling and screened 5,237 litigation documents using keywords, including medical errors. Second, 2,536 samples were retained through systematic random sampling, and 549 irrelevant cases were deleted after manual reading. Finally, three clinicians from different medical departments selected 329 incidents related to orthopaedics for further analysis, according to the description of the lawsuits. Three other professional orthopaedists evaluated the patients’ ages, affected sites of medical errors, and adverse consequences.

**Results:**

The greatest number of medical errors was observed in the joints (30.43%) for all orthopaedic patients. However, adult patients (aged 18–60 years) were most susceptible to errors in the extremities (30.42%). A higher rate of complications was associated with a higher rate of morbidity/mortality for the corresponding patients. Medical errors correlated with complications occurred in the following sites: joints (15.38%), extremities (12.50%), spine (16.95%), multiple sites (15.38%), and hands and feet (14.81%). In addition to surgical errors, over 10% of all orthopaedic patients experienced missed diagnoses. The incidence of insufficient adherence to informed consent obligations was 13.5% among adult patients and was much higher in paediatric and older adults patients. When orthopaedic patients suffered from medical technical errors, iatrogenic mortality/morbidity would decrease by 0.3% for one unit increase in age.

**Conclusion:**

Dividing patients into different ages demonstrated diverse results in terms of medical errors and affected sites. Negligence in diagnosis and examination can be fatal factors that endanger safety, and complications may cause morbidity/mortality. When patients suffered from technical errors, age is inversely proportional to mortality/morbidity. Special attention needs to be paid to technical errors in the younger older adults population (60–64 years old), which has inspired implications in promoting aging and public health.

## Introduction

As fears about the adverse impact of medical errors in the orthopaedic department grow, issues related to patient safety in surgical specialties have garnered international attention. Previous studies illustrated that the clinical departments with the most surgical accidents are trauma and orthopaedics, of which 30.1% of the incidents cause iatrogenic injuries to patients (1). In addition to higher morbidity and mortality, medical errors might lead to unnecessary health costs for patients (2), which induce increased medical litigation and insurance premiums in the orthopaedic department (3). The grim reality of the present includes the untoward search for insurance companies that are willing to take the risk of making monetary claims due to medical malpractice (4). Moreover, defensive medical behaviours are gradually increasing, and rising medical costs have resulted in the overuse of medical resources without benefiting patients (5). Hence, exploring the causes of medical errors in orthopaedic departments has become the focus of solving the aforementioned problems.

Most researchers blame surgery for medical errors in orthopaedic departments. At present, surgery has been categorized as a “very unsafe” industry, which could increase the hazard of adverse events in the clinical stage (6, 7). Meanwhile, other scholars subscribed to the view that failed teamwork in the operating theatre became an inevitable cause of medical errors (8). But some scholars hold different opinions that medical errors in the orthopaedic department may be related to humanistic factors including inadequate informed consent and invalid communication between physicians and patients during the preoperative period (9, 10). This leads us to question whether the medical errors in the orthopaedic department result from the surgeries, failure to embrace sufficient communication between physicians and patients, or a combination of both, which would further advance the preventive measures implemented in clinical practice. The aforementioned academic debates may reveal, to some extent, that research on patient safety in orthopaedic departments is still necessary.

Existing studies have preferred to regard orthopaedic patients as a whole and primarily concentrate on the adult population. Previous scholars have proven that the spine is the affected site with maximum errors (11). But according to a survey by the American Academy of Orthopaedic Surgeons (AAOS), the knees, fingers, and hands have the highest probability of medical errors, whereas the spine has the lowest probability (12). Despite wrong-site surgery being the error that elicited the most successful litigation (13), there are conspicuous disparities among different patient groups. A study of paediatric patients demonstrated that the most common reason for medical litigation was missed or incorrectly diagnosed injuries, and 44% of these were upper limb injuries, mainly fractures around the elbow (14). However, hip fractures are more severe in the older adults population, resulting in substantial morbidity and mortality (15). We believe that existing research lacks empirical analyses from the perspective of age, which may explain the paradoxical conclusions previously drawn by scholars. Further, if patients are not compared by different age groups, most countermeasures may be invalid in addressing adverse events, especially in the orthopaedic department.

To sum up, we proposed following research questions. What are the characteristics of orthopaedic patients in different age groups when suffering from medical errors? Which sites are more prone to be affected by medical errors among different age patients in the orthopaedic department? Have medical errors caused iatrogenic mortality or morbidity among orthopaedic patients? To answer these questions, we utilized the available medical litigation to systematically sort the medical errors, prone sites, and adverse consequences of suffering by paediatric, adult, and older adults patients in the orthopaedic department. This study contributes to the existing literature in three aspects. Firstly, the comparative analysis among patients can fill the academic gap in existing research to enrich the comprehension of orthopaedic medical errors. Secondly, grouping comparisons from an age perspective can provide orthopaedic practitioners with more targeted preventions. Third, grouping patients by age will provide more detailed comprehensions of medical errors in paediatric and older adults orthopaedic patients, which will play an imperative role in promoting aging and public health in particular.

## Materials and methods

### Study design and sample

Research on patient safety in China habitually lags behind that in other countries due to a lack of data, as the medical error collection system has not yet been constructed. However, China Judgments Online (CJO) provides a new approach to the study of medical errors. According to the provisions of the Supreme People’s Court of the People’s Republic of China, except for particular circumstances stipulated by the law, legally effective judgments, rulings, and decisions should generally be available on the CJO website. Hence, it is possible to retrieve litigation documents related to medical errors on the public CJO website for research without ethical approval. The Ethics Committee of Jinan University (NO. JNUKY-2021-035) approved this study and we confirm all methods were performed in accordance with the relevant guidelines and regulations.

**Figure 1:**
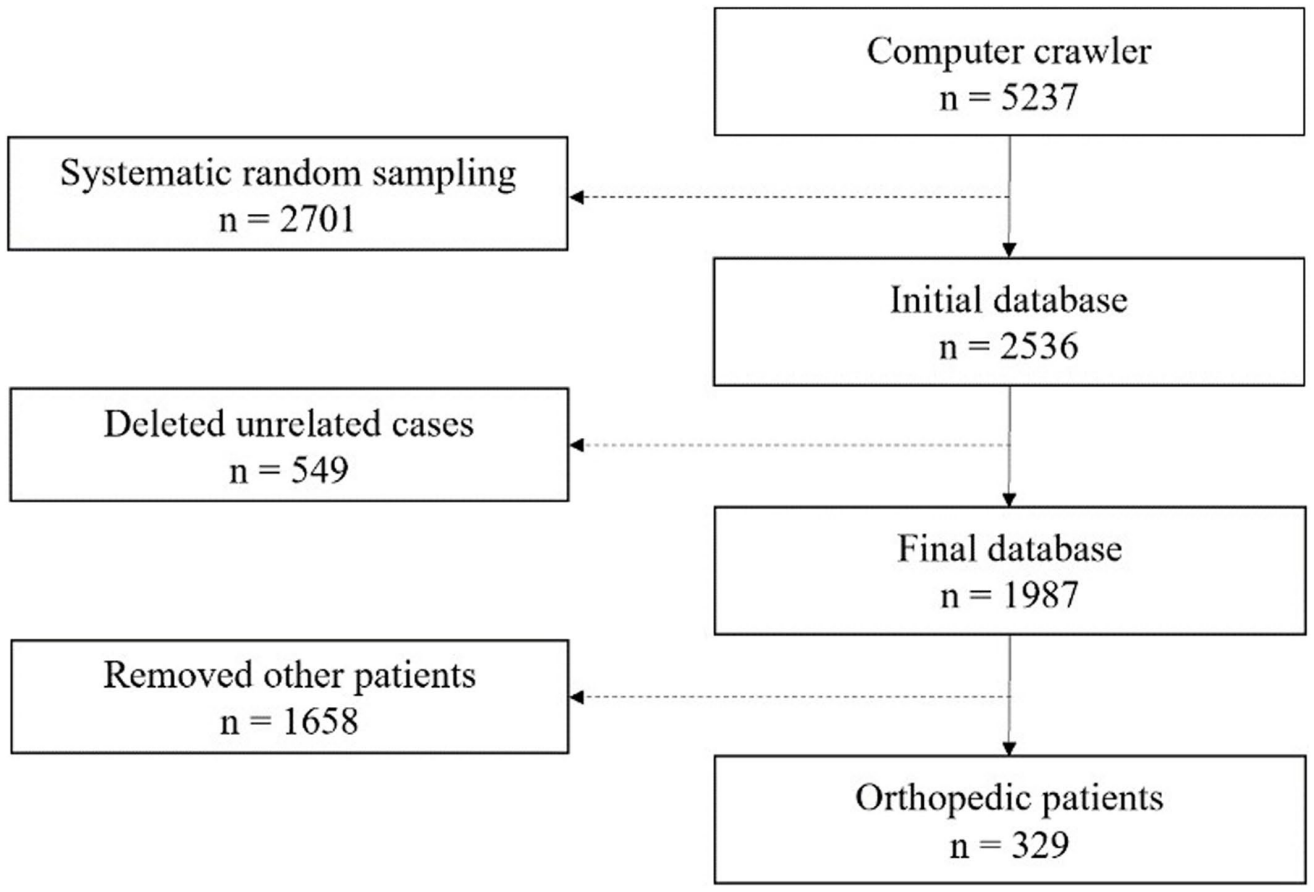
Process of sample selection.

The samples used in this study were selected in the four steps detailed in Figure 1. First, we utilized keywords with regard to medical errors and adverse events to computer crawl on the CJO website and retrieved a total of 5,237 litigation documents. Second, we eliminated 2,701 documents through systematic random sampling due to the limited research funding and the high cost of manual reading, including some samples with missing texts as well. Third, we removed 549 documents that were unrelated to medical errors through manual review. These unrelated documents do not cover the content of medical errors from orthopaedic department and are inconsistent with the subject of this study. Finally, we invited three clinical physicians to identify the medical errors and specialties in 1,987 litigation documents. We obtained 329 orthopaedic samples after removing 1,658 documents from other specialities.

### Data collection

For the coding of medical errors and departments, we referred to the definition and classification criteria in the existing literature. We defined medical error as an act of omission or commission in planning or executing that contributes or could contribute to an unintended result (16). Combing another study on medical errors in China (17), we identified the types of medical errors and the classification of medical departments. The specific classifications of the medical errors and departments are detailed in Appendix 1. Initially, two clinical physicians independently reviewed litigation documents and judged the medical errors and specialties involved. A third doctor reread any inconsistent results and determined a final judgment. We then retained 329 cases that appeared in the orthopaedic department for further coding. To obtain accurate data, we invited three professional orthopaedists with more than 10 years of clinical experience to conduct a second batch of coding. According to the text in the litigation documents, two orthopaedists coded the patient information, including sex, age, type of surgery, affected sites of medical error, mortality, morbidity, and complications.

To analyse the affected sites of medical errors among patients of different ages, we divided them into three groups according to age range: paediatric patients (aged ≤18 years), adult patients (aged 18–60 years), and older adults patients (aged ≥60 years). Based on whether the patients underwent surgery, we classified the surgery types into surgery and non-surgery groups and further divided surgery into emergency surgery and selective surgery. Combining the directories of orthopaedic subspecialties in Chinese clinical practice and the classification of orthopaedic surgical sites in existing literature (18), we grouped the sites affected by medical errors into the following seven categories: joints (excluding extremities and spine), spine, pelvis, hands and feet, limbs, multiple sites, and other. Finally, a third orthopaedist reviewed the disparate results and independently provided the final judgment. All information about the patients is anonymous.

We utilized LOGIT regression to investigate the association between the iatrogenic mortality/morbidity and the age of the patient. The independent variable was the age of the patient, which was extracted from the patient information in the litigation documents. The dependent variable was whether iatrogenic mortality/morbidity occurred. We assigned a value of “1” to iatrogenic mortality/morbidity, “0” to no iatrogenic mortality/morbidity. The control variables included gender, whether multiple departments were involved in treatment, hospital class, and affected site.

## Results

**Figure 2:**
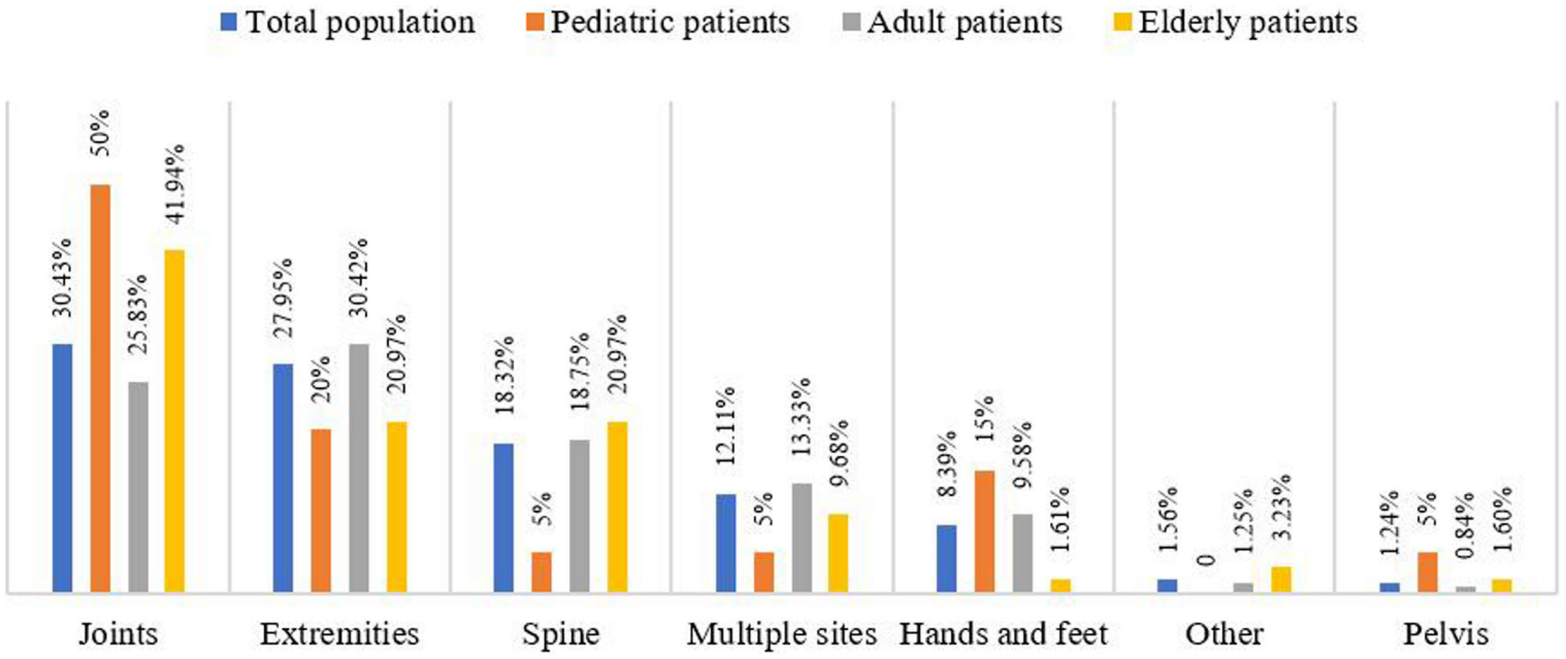
Affected sites of medical errors among different population.

In terms of the total patient population, the joints, extremities, and spine were the three sites with the highest rates of medical errors (Figure 2). However, there were apparent discrepancies in the affected sites after subdivision into different patient groups. For example, joints were the most frequently affected sites in paediatric and older adults patients, but adult patients were more prone to medical errors in the extremities. Although the incidence of medical errors in the spine increased with the age of the patients, the hands and feet showed diametrical results, as the proportion of medical errors was highest in paediatric patients.

**Figure 3:**
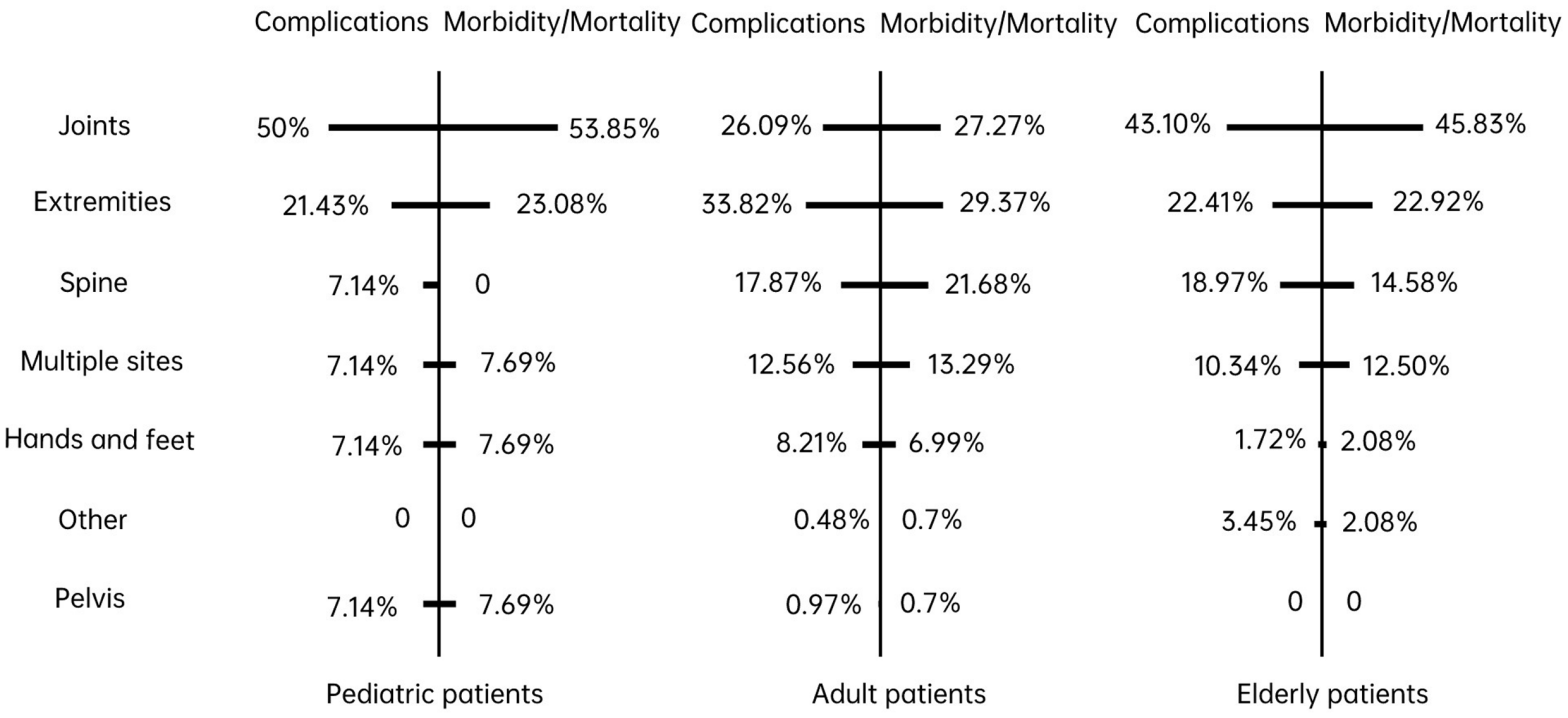
Complications and morbidity/mortality of affected sites among different patients.

As shown in Figure 3, different affected sites may cause different degrees of harm to patients in diverse groups. Furthermore, even though we cannot infer that complications were the fundamental reason for morbidity/mortality of patients, as 7.14% of paediatric patients had medical errors in the spine, but none suffered morbidity/mortality, we realized that there was a high association between complications and morbidity/mortality within different affected sites. Specifically, a higher incidence of complications was associated with a higher rate of morbidity/mortality in the corresponding patients. Half of the paediatric patients suffered from complications due to medical errors in the joints, and morbidity/mortality accounted for 53.85%. It is worth noting that similar circumstances appeared in adult patients with medical errors in the extremities and in older adults patients with medical errors in the joints.

To better understand the relationship between medical errors and affected sites, we calculated the three most frequent types of medical errors at different sites. The first column in Table 1 is the affected sites where medical errors occur, and the number in the brackets is the proportion of errors in each site. The results in Table 1 show that medical errors correlated with complications, especially post-operative complications, arose in all included sites: joints (12.77%), extremities (12.50%), spine (16.95%), hands and feet (14.81%), and multiple sites (15.38%). In addition, insufficient implementation of informed consent obligations attached to physician-patient communication was the foremost type of medical error in extremities (19.32%), and multiple sites (28.21%). Missed diagnoses were found in joints (9.57%), extremities (12.50%), spine (10.17%), and multiple sites (10.26%), compared with other sites. Although we cannot assert that there are causalities between missed diagnoses and the four aforementioned affected sites, missed diagnosis is still a vigilant signal.

**Table 1:** Three most frequent medical errors in different sites.

Sites	Medical error 1	Medical error 2	Medical error 3
Joints (30.43%)	Complication of post operation (12.77%)	Missed diagnosis (9.57%)	Failed to cure the protophathy (8.51%)
Extremities (27.95%)	Insufficient implementation of informed consent obligations (19.32%)	Missed diagnosis (12.50%)	Complication of post operation (12.50%)
Spine (18.32%)	Complication of post operation (16.95%)	Missed diagnosis (10.17%)	Failed to cure the protophathy (6.78%)
Multiple sites (12.11%)	Insufficient implementation of informed consent obligations (28.21%)	Complication of post operation (15.38%)	Missed diagnosis (10.26%)
Hands and feet (8.39%)	Improper time of surgery (14.81%)	Complication of post operation (14.81%)	Failed to cure the protophathy (11.11%)
Pelvis (1.24%)	Delay of timing during treatment (50%)	Intraoperative damage of tissues and organs (25%)	Improper treatment of postoperative complications (25%)

All orthopaedic patients are susceptible to errors in medical technology; however, these errors are not restricted to surgery. Medical errors in diagnosis and examination also impair orthopaedic safety, especially in the paediatric population. As shown in Table 2, missed diagnosis (3103) was not the most continual error but was experienced by over 10% of all orthopaedic patients. Errors in the treatment plan (3303) severely impacted paediatric patients, and the incidence was four times higher than that of adults. The prevalence of post-operative complications (3609) in adult and older adults patients was 13.92 and 13.56%, respectively. However, among adult patients, the rate of insufficient implementation of informed consent obligations was 13.5%, which was much higher than that of paediatric and older adults patients.

**Table 2:** Top ten medical errors of patients in different age groups.

Rank	Paediatric patients	Percent	Adult patients	Percent	Older adults patients	Percent
1	3,303	15%	3,609	13.92%	3,609	13.56%
2	3,203	10%	6,202	13.5%	3,103	10.17%
3	3,103	10%	3,103	11.39%	3,203	8.47%
4	3,609	10%	3,607	5.49%	3,607	8.47%
5	3,305	10%	3,608	4.64%	6,202	8.47%
6	6,202	10%	7,103	4.22%	3,301	5.08%
7	3,301	5%	3,301	3.8%	3,608	5.08%
8	4,105	5%	3,303	3.38%	3,101	3.39%
9	3,603	5%	4,104	3.38%	3,305	3.39%
10	3,607	5%	3,101	2.95%	4,104	3.39%

Please refer to the Appendix 1 for the specific types of medical error that the code indicates to.

Additionally, 4.22% of adult patients challenged informal writing or modification of medical records (7103), which was remarkably different from the other two groups. Although the insufficient implementation of informed consent obligations correspondingly threatens the safety of older adults orthopaedic patients, insufficient inspection (3203) was nearly 3.3 times higher than that of adults. In summary, there are discrepancies in medical errors among different patients. Medical technical errors and medical humanistic errors are the two chief foundations of litigation in orthopaedic departments.

Given the disparities in the harm of medical technical errors and medical humanistic errors to different age groups of patients, we performed regression analysis for patient’s age and iatrogenic mortality/morbidity. As shown in Table 3, there is no significant relationship between age and the iatrogenic mortality/morbidity for medical humanistic errors. However, for medical technical errors, iatrogenic mortality/morbidity decreases with age. Specially, the iatrogenic mortality/morbidity decreased by 0.3% for every year increase in age. This finding reveals that as orthopaedic patient’s age increase, the damage caused by medical technical errors decreases.

**Table 3:** Relationship between age of patient and iatrogenic mortality/morbidity.

	(1) Medical humanistic errors	(2) Medical technical errors
	Iatrogenic mortality/morbidity (OR)	Iatrogenic mortality/morbidity (OR)
Age	0.985	0.997^**^
	(0.015)	(0.01)
Control variable	YES	YES
_cons	664	4.407
	(5363)	(4.702)
pseudo *R*^2^	0.32	0.1
*N*	33	132

The OR is also known as the “odds ratio” or “relative risk.” In the logistic regression model, p = P(y = 1|x) and 1−p = P(y = 0|x). The term “p/(1−p)” is referred to as the odds ratio. Standard errors in parentheses. ^**^ *p* < 0.05.

## Discussion

Through quantitative analysis of medical litigation documents, we concluded that the joint was the most prone to medical errors. However, after adding the variable of age, diverse patients manifested dissimilar characteristic trends in the affected sites. Previous findings have shown that the majority of deaths or severe permanent injuries coexist with the spine in all orthopaedic patients (18). But we recognized that paediatric and older adults patients were more likely to experience medical errors in the joints, which is akin to the previous conclusion that operative procedures on joints resulted in the most claims in paediatrics group (19). Hence, standardize care processes have been regarded as the best tool for implementing safety culture in paediatrics (20). While the extremities were the primary sites for medical errors in adult patients, revealing that affected sites with the most medical errors were different after dividing the patients into groups according to age. This discrepancy suggests that attention to certain medical error sites should be allocated separately to different groups rather than considering orthopaedic patients as a whole. This will not only provide more detailed countermeasures for preventing medical errors but will also effectively improve patient safety in different populations.

A broad consensus has been reached that surgical safety should be a substantial global public health issue (21), but it is worth considering the adverse consequences of medical errors in other clinical stages. Most medical errors are linked to medical technology (12, 22); however, medical errors in the diagnosis and examination stages seem to be neglected in the orthopaedic department. Diagnosis and examination are the two prerequisites for successful surgery, but existing studies have overemphasized improper procedures during surgery (22) while disregarding precise diagnosis and sufficient examination. We concluded that over 10% of orthopaedic patients suffered from inaccurate diagnosis, and the impact of errors in treatment plans for paediatric patients and insufficient inspection of older adults patients were more serious than those of adult patients, which may have been formerly overlooked by scholars. Although orthopaedists place more emphasis on surgery, accumulating rich expertise and developing skilful diagnostic abilities are imperative in practice (23), especially when providing clinical receptions for paediatric patients (24).

There are disparate emphases on how to rectify medical errors in orthopaedic departments. We are not opposed to the indispensable role of surgical checklists in promoting patient safety (25, 26). A free and simple intervention, such as a two-lesson training, significantly stimulated the correct use of the surgical safety checklist (27). Safety guidelines in anaesthesia and obstetric departments have successfully achieved the goal of promoting patient safety (28). The implementation and regular use of surgical checklists may also become important elements in orthopaedic safety programs (29). However, the surgical safety checklist has been finitely applied in orthopaedic departments in practice, with 65.3% of Brazilian orthopaedists unaware of this protocol (10). In addition, the FDA created an analogous program called MedWatch to aid healthcare professionals in reporting adverse events, which aimed to help orthopaedic surgeons protect their patients (30). Other scholars believe that shifting the culture surrounding medical errors from blame to understanding and prevention could accomplish mutual benefits for patients and surgeons in orthopaedic departments (31).

Additionally, it is equally vital to enhance physician-patient communication to prevent additional medical errors in the orthopaedic department. Generally speaking, verbal aggression toward the physicians or between patients has been the most reported events (32). Despite having identified the insufficient implementation of informed consent obligations as a challenge for orthopaedists (33), further analysis indicated that this challenge may be more frequent in adult and older adults patients, especially in the adult population. This academic contribution differs from existing conclusions. However, some countermeasures and suggestions provided by previous research seem to align with this study. Therefore, we emphasize that, in addition to the necessary attention given to the surgical stage, treatment, diagnosis, and physician-patient communication might equally require more consideration.

Finally, orthopaedists must be alert to medical errors associated with complications, which, in addition to leading to medical claims (34), may lead to further morbidity and mortality (35). Owing to the lack of rigorous experimental data, we cannot provide scientific answers to the causality between complications and morbidity/mortality. However, we observed positive correlations between complications and morbidity/mortality, which might provide innovative insights for promoting orthopaedic patient safety in the future. Most complications in orthopaedic surgery can be dealt with adequately, provided they are anticipated with implemented risk-reduction strategies (36). Hence, we should prioritize addressing medical errors by combining the age and affected sites of patients in clinical practice. Additionally, the COVID-19 pandemic has given to the spread of telemedicine in the orthopaedic field (37), which might be an effective approach to improve the patient safety in the orthopaedic specialty. Previous interventions might be less effective, as they are slightly generic and exceedingly concentrated on the operation stage.

## Conclusion

Subdivision of orthopaedic patients into different groups based on age demonstrated entirely dissimilar results in both affected sites and medical errors. In addition to surgery, negligence in diagnosis and examination is also a fatal factor endangering patient safety. Complications may cause morbidity or mortality among different orthopaedic patient groups, but the underlying mechanism remains unclear. Patient age and affected sites should be integrated to discern the medical errors that may occur in the orthopaedic department and prevent adverse events in clinical practice accordingly. Additionally, special attention needs to be paid to medical technical errors in the younger older adults population because they have higher iatrogenic mortality/morbidity in comparison to senile patients (≥ 65 years old). This finding has inspired implications in promoting aging and public health.

### Limitations of this study

Due to the limited samples of paediatric patients, the analysis seems to be insufficient for this population. More patient demographics were not included in the litigation documents; hence we were unable to perform more quantitative analysis in this dimension. Similarly, we were unable to explain the causal mechanism between complications and morbidity/mortality. However, the database in this study has been the best material to study patient safety in China so far. The conclusions could promote health for patients and guide clinical practice for medical staff as well.

## Data availability statement

The original contributions presented in the study are included in the article/Supplementary material, further inquiries can be directed to the corresponding authors.

## Ethics statement

The studies involving humans were approved by the ethics committee of Jinan University waived the need of informed consent. The studies were conducted in accordance with the local legislation and institutional requirements. Written informed consent for participation was not required from the participants or the participants’ legal guardians/next of kin in accordance with the national legislation and institutional requirements.

## Author contributions

PL: Conceptualization, Funding acquisition, Investigation, Writing – original draft. JC: Methodology, Resources, Writing – review & editing. YY: Data curation, Methodology, Software, Writing – original draft. HZ: Conceptualization, Data curation, Supervision, Visualization, Writing – review & editing.
